# Criminal Assault by a Dentist

**Published:** 1858-12

**Authors:** 


### CRIMINAL ASSAULT BY A DENTIST.

We copy from the "Montreal Herald" the following part ticulars relating to the trial of a well known dentist of Montreal, who has been found "guilty of an attempt to commit a rape, with a recommendation to mercy," at the Criminal Term, now being held in this city. We forbear, for the present, making any remarks on the case:—

"Dr. John Horatio Webster was placed at the bar charged with having, on Wednesday last, the 22nd September, committed a criminal assault on Louisa Chandler, wife of Mr. James Nichols, of this city.

The complainant, Mrs. Louisa Nichols, was examined by Mr. Monk, Q. C.:—I am the wife of Jas. Nichols, and reside in Fortification Lane. On Wednesday last I went to the Surgery of Dr. Webster in Notre Dame Street; I wished to have the pattern of my mouth taken. I went to the surgery about half-past ten o'clock. Before I entered the surgery I remained for a time in the waiting-room. There were two ladies in the surgery when I first entered the waiting-room. The prisoner was called in to speak to me; I then gave him some gold leaf, which he was to use in operating on my teeth. At the same time, I asked him if he could look at my mouth then; he told me to wait a little, and he would be glad to examine it. I remained accordingly, but not more than five minutes elapsed till he again came into the waiting-room, to show the two ladies down stairs. A gentleman then came in. Dr Webster then asked me to wait about half an hour longer, till he would operate upon this gentleman. I consented. After this, three ladies and a gentleman came in; they were friends of Mrs. Webster. Shortly after, the gentleman already mentioned, left the surgery. The Dr. told me to enter; in the meantime he went into the waiting-room; the prisoner followed me in a few minutes. I stood close to the dental chair, and he examined my mouth. I asked him if he though it necessary to remove a stump of an old tooth. He said he thought it would be well to take it out; the new teeth would do for life; the stump only cause irritation of the gum. I said if it were necessary to take it out it would be better to have no new teeth. He replied in the negative, and said I ought to take chloroform. He said also, that, under the use of chloroform, he could extract it in five minutes. In the operating chair Dr. Webster administered the chloroform. I

did not consent immediately to inhale the chloroform. About five minutes elapsed from the time he proposed till I took the chloroform. During the interval I told him chloroform would make me sick. He replied that I had better take it; it would not influence me very long. This was all that was said. He applied the chloroform by means of a cloth. I took chloroform about four months before, and what he administered was the same substance. I did not become immediately insensible. It was about half-past ten o'clock when I went into the waiting-room; about a quarter to twelve when I entered the surgery. After taking the chloroform, the first thing I remembered was his bringing the forceps to extract the tooth. I said to him, "Have you not taken it out yet?"—he said "No;" he then administered some more chloroform. I did not perceive how he administered the chloroform, but I felt myself inhaling it. I then became unconscious. At length I became partially conscious. I saw I was not in the chair, but on the sofa; the sofa was not very far from the chair. When I become conscious I saw that the prisoner was sitting along with me on the sofa. When I first became conscious, I felt the pressure of the prisoner's body; (the witness then went on to state to the Court—an improper interrogation he had directed to her—the indications by which she was aware that the prisoner had taken criminal liberties; ultimately she said she heard a bell ringing.) I then threw my limbs off the sofa, and said to the prisoner, "I wish you would send for my husband." I again became unconscious, and felt very sick at the stomach. The next circumstance that I recollect was that the prisoner was sitting beside me on the sofa, and had his hand in an improper position, and that he had placed mine in the same. I then asked him to get a cab to take me home. He said I would be unable to proceed. He then said he had got the wax hot to take an impression of my mouth, and that that was the time to take it. I said nothing. He then carried me from the sofa to the chair, but I was still partially unconscious. He brought the wax, and took the impression of my mouth. While doing this I had not quite recovered my consciousness. When he had taken the impression of my mouth, the bell rang, or some one called, and then he left me. I sat still and vomitted considerably; at length I got upon my feet; the prisoner opened the door communicating between the waiting-room and the surgery, and allowed me to pass out. He following behind me coming down stairs. Passing through the waiting-room, I saw that there were three gentleman there ; on leaving, he told me he would have the teeth ready by Friday, if not, he would send me word. I then went home. All this occurred on Wednesday, the 22nd of this month. The second time I come to consciousness, I felt the weight of he prisoner on me. I feel confident of this fact. [Witness here proceeded to detail her reasons for this assertion. They were unfit for publication.] She went on I have no doubt at all that the prisoner was in this position; but at the same time I was not sufficiently conscious to perceive the state of my clothes. He did not extract the stump of my tooth; it is yet in my mouth. When he gave me the chloroform it was with the intention of drawing this stump. He assigned as a reason for not extracting the stump, that he had not gas enough in the chloroform. He administered the chloroform three times. I have already stated, that during the period of consciousness I was lying on the sofa, he sitting on the sofa beside me, and his hand on a certain part of my person. I have now stated all that I recollect. On arriving at my own house, I spoke to my husband. I told him that I would not again go to Dr. Webster's; because I thought he was a nasty, dirty old fellow. When I perceived the prisoner in the condition I have already mentioned, I recollect pushing him with my hand as well as I could. When I went home I perceived my under-clothes were very wet. I became aware of this circumstance before I left the prisoner's office. I thought it was my own urine, and attributed it to the fact that I had been greatly pulled about. When I first took the the chloroform I was sitting on the dental chair ; when I became conscious I was on the sofa. I do not know how I was removed.

Cross-examined by Mr. Devlin.

About four months ago I took chloroform. It was administered by the prisoner. I was then getting my mouth prepared for a new set of teeth. I took no chloroform before or since until this last occurrence. The first time I took it I was accompanied by my little daughter. My stomach then grew sick; but my clothes were not wet at that time. On Wednesday last, when the prisoner brought me into the surgery, he did not lock the door communicating between it and the waiting-room. The door might have been partially open. When I was in the waiting-room, Miss Webster was there; she was neither reading a book nor playing the piano: she was talking to a friend. When I entered the dental chair, I took of my bonnet and shawl and placed them on the sofa. The prisoner told me to take them off. While I was disrobing he went to get the chloroform. At the time I went into the dental chair I heard no voices in the waiting-room. There is another door leading from the surgery into the laboratory or workshop; it was not wide open, but was partly open like the other. It was not open enough to enable me to look into the workshop. I saw no young man in the workshop when I went into the surgery; but when I was prepared to come away I did. The person I then saw was talking to a little boy; the taller of the two was standing opposite the door at the far end of the workshop; the little boy on a bench, the tall young man was standing there talking, I suppose; I don't know what he was doing. I don't recolleet if I spoke to the tall young man.

Rowland Webster the young man alluded to was brought forward, and recognised by the witness. A boy was brought forward also, but she was not certain of his being the boy to whom she alluded.

Cross-examination continued: I might have heard the tall young man ask me if I were sufficiently strong to proceed home. I did not hear this young man call the prisoner from the waiting-room to me before I left; but he might have done so. Before I left, the Dr. was called into the waiting-room; I was in the dental chair at that time. I do not remember having asked the Dr. to give me chloroform. Both these doors were partly open when I sat down on the chair. I think what relieved me of the weight of his body was the ringing of the bell. At the time I was partially unconscious. I did not feel this pressure a second time; I did not feel the weight more than a second; he was then called away by the bell I think; the weight was instantaneous; I was unconscious from the time I entered the chair till I felt myself upon the sofa; at least I was partially so. I felt the prisoner's hand in an improper position upon my person, but I did not see it. I did not see my own hand in a similar position on his. The first thing I saw on opening my eyes was the prisoner adjusting his clothes. I felt but did not see him sitting on the sofa beside me. I swear that the person who sat beside me on the sofa was not the tall young man.

Mr. Devlin—By what means can you tell that?

Witness—Must I answer this question?

Mr. Devlin—Yes.

Witness—I can say without doubt it was not the young man; because I heard the Dr. speak. I heard him speak when he was addressing to me improper interrogations; but I think I said nothing except wishing that my husband were there. With a great effort I was enabled to say as much as this. I made no effort to shout to any one in the next room. [The Witness gave this answer after much hesitation.] When I said to the prisoner that I wished Mr. Nichols was there, I was lying on the sofa on my side. I did not open my eyes. I made an effort to get off the sofa, and threw my legs out; he had got off before this to answer to some one who called or rang for him. I did not then call for assistance. When the prisoner then left me, I neither saw the tall young man nor the boy. Passing out of the waiting-room, I did not tell the gentleman there that I had been abused. I spoke to no one in the house about what had happened. I made no promise to come back on Friday. I was carried back into the dental chair a second time, but I did not see the young man or boy. Tne sofa on which I was lying is within a few feet of the workshop door, but I never measured the distance. When I came home I told my husband that the prisoner was a dirty, nasty old fellow. This occurred on Wednesday. My husband wrote to the prisoner on the same night. On Saturday we laid the facts before Mr. Coursol. I had communicated with a lady friend of mine, Mrs. Laurie, about the abuse I received. I don't know why my husband and I did not immediately go before a court instead of waiting till Saturday. It was the Doctor, and not the young man who showed me down stairs. When I first visited Dr. Webster in April last this young man, Webster, was present watching the effect of the chloroform administered. I swear that I had no conversation on Wednesday last with the young man Rowland Webster. On the day in question the prisoner did not say that he was unwell. I was forty-one years of age last August. In the surgery of the defendant no one assisted me to dress. I reached home; I remained about the house all day. I had no friends at my house that evening. During the interval between Wednesday and Saturday my husband and I were talking about the abuse I received; we were talking of it all the time. I told some friends. When I took chloroform in April last, Dr. Webster told me that it caused me to cry and fight. I don't recollect if I were told that it produced any other peculiar effect. Last Wednesday, when talking these circumstances over with my husband, had a perfect recollection of all that occurred. I did not fully remember everything till the next day, Thursday. I sent my under-dress to a Doctor for examination; the name of the medical man is Dr. Fenwich. The articles were a chemise and a cloth; the latter I wore for a certain object. I did not take it off in the surgery, but the prisoner managed to place his hand underneath the cloth, though it was wet and soiled, but not removed from the place where I myself put it. I have not heard what is the result of the examination of the cloth. When sent to the Doctor it was not much soiled. When on the sofa, this cloth was tied on in the same position as I had placed it in the morning. I said that I believed the prisoner had effected his purpose, though this cloth was tied on. I told this to my husband.

Mr. Devlin—Did you not swear that the cloth, when you got up off the sofa, was in the same place as when you lay down?

Witness—Of course it was; where else would it be?

Cross-examination continued—I believe he violated my person.

Mr. Devlin—How long did he take to effect his purpose?

Witness—I don't know.

Mr. Devlin—Did you not swear that the pressure was instantaneous.

Witness—Only at that time.

Court—You may go down.

James Nichols, husband of the last witness, examined by Mr. Monk, Q. C.

Witness—My wife told me Dr. Webster was a villain. She explained to me the reason of this assertion, but not till about two o'clock in the morning; she then told me he had violated her person: she told me he was aided in his design by chloroform. I saw when she came in that she was laboring under great excitement. On Friday morning having been made acquainted with the details, I laid the case before Mr. Rose.

Cross-examined by Mr. Devlin—When she came home I gave her some brandy and water to revive her; she eat a little rice-pudding afterwards.

Mr. Devlin—I now leave it to the court if it is necessary to go upon a defence. I think the Court will say there is no evidence to lay before the Jury on part of prosecution.

Mr. Monk—What has been brought out in the cross-examination is, I think sufficient.

Court—Penetration has not been proved; and the Jury can not go upon the belief of a woman while under the influence of chloroform. You will therefore, Mr. Devlin, take up the case as if it were one of attempt at assault; the question of rape must involve penetration or emission, but neither has been proved. So you need not address the Jury as if your client were indicted for capital felony; that has not been at all proved.

Mr. Tate, architect, examined by Mr. Devlin—in company with Mr. Brown I examined the rooms of Mr. Webster. It would require some amount of force to shut the door leading from the laboratory to the consulting-room; the lock was on that side of the door which was within the laboratory.

Mr. Monk objected to evidence of description.

Mr. Devlin said he wanted to prove by the plans of the apartment he then had in court that the door between these rooms was so situated that the person working in the laboratory could see from his bench what was taking place in the surgery.

(The plan of the apartments were sent up to the court.)

Court—Have you got the measurement of the rooms.

Witness—Yes. (The witness was here directed by the Court to take the plan of the apartments to the Jury, and explain their length and dimensions, etc., with the position of the doors, but not to say anything about the arrangement of the furniture.)

The witness complied.

Examination continued—If the door were partially opened, any one working in the laboratory, unless he turned his back, must see what is going on in the surgery; and hear also, as the place is so small.

By Mr. Monk—I examined the apartments yesterday. I don't think any of the furniture was removed.

Rowland Webster examined by Mr. Devlin—I am a cousin of the prisoner's and have been engaged with him learning his profession, since December last. In the month of April last, I saw the prosecutrix in the surgery; she came to have teeth extracted. On Wednesday last I saw her in the same place. I was melting gold that day; using a forge for the purpose. I saw the Dr. give her chloroform. He then made an attempt to extract the tooth. I saw her when she was taken from the chair and when she lay down on the sofa. This was after twelve. When she lay down the Dr. went into the parlor. There were some persons there; but I don't know who they were. From my place in the laboratory I could see the sofa. I had no conversation with her till she was about to go home. She asked me if I thought she were strong enough to go home. I then called the Dr. from the parlor. He told her she had better lie down again if she did not feel strong enough to go home. The Dr. went back to the parlor; she then put on her bonnet, and the Dr. came in again and told me to see her out. I swear I was the person who escorted her down stairs. From the time she came in till she left, I saw nothing occur to her. Since last December, the doorbetween the laboratory and surgery was never closed; there is no lock nor key for it. There where people in the parlor when she came in and when she went out. There was a gentleman in the chair before she occupied it. From the time she came in till she left, I swear she was not insulted by the prisoner.

By Mr. Monk—I am there every day. The door between the laboratory and surgery is always open. Mrs. Nichols came into the surgery about half-past eleven. That was the first time I saw her that day. There were some patients inside when she came. I do not know when she arrived. During the time Mrs. Nichols was in the surgery the door was about half open. I was in the laboratory all the time, for I had to keep the fire of the forge alive all day. I only left the laboratory when she prepared to go home, about half past one o'clock. I was melting gold in the forge during the time she was in the surgery. I began this labor at ten o'clock, and worked at it for about three hours; then I began to melt zinc till three o'clock the chloroform was administered about ten minutes after she entered the dental chair. She then lay down upon the sofa for the remaining hour and forty minutes. I did not see her or watch her all the time she was on the sofa. When she walked from the chair to the sofa she was under the influence of chloroform.

Court—Is it customary to give chloroform in the presence of witnesses?

Witness—Sometimes.

Court—Did you see it administered?

Witness—Yes.

Court—What is the longest time you have found females remain under the influence of chloroform?

Witness—An hour.

Court—Where are they placed?

Witness—On the sofa.

Court—How do they get there? Does the prisoner carry them?

Witness—He leads them, or they walk themselves.

Court—Does he leave a patient to remain an hour under the influence of chloroform, without looking at the state in which they are?

Witness—I don't think he does, sir.

Court—Would he see them four times in the course of an hour?

Witness—Yes.

Court—How often did he visit Mrs. Nichols during the time she was under the influence of chloroform?

Witness—He did not visit her at all.

Court—Did you ever know any other female remain an hour and a half under the influence of chloroform and know the patient to be unattended?

Witness—No.

Court—Why was it done in this instance?

Witness—I don't know.

Court—You attend upon women who take chloroform to have teeth extracted?

Witness—Sometimes.

Court—You may go down.

Mr. Jas. Goodrich examined by Mr. Devlin—I once took my wife to the prisoner, and she inhaled chloroform to undergo an operation. During the remainder of the day she was under the strongest impression that she had been taken into a room and violated, and continued to hold the conviction, though I told her I was by her side all the time and had my finger on her pulse.

Mr. Monk— She thought she was violated ?

Witness—Yes.

Mr. Monk—And whom did she accuse ?

Witness—If the truth must me told she supposed it was Dr. Webster; and it was with great trouble that I disabused her mind of the idea. She was under the influence of chloroform for about ten minutes.

Dr. W. Nelson examined—I have known the defendant for 16 years. I was about the first in Montreal to use chloroform. It produces the most contrary effects in different individuals. I once operated on a woman who had a tumor. I got the loan of an apparatus to administer ether; I received it from Dr. Webster. The patient took ether, and I removed a tumor of seven pounds weight. The woman for two days held the opinion, though many of her neighbors were witnesses of the operation, that I abused her. The witness related one or two other incidents to shew the peculiar effect of ether and chloroform. The Dr. then went on to state his high opinion of Dr. Webster ; how he had recommended him as a dentist to some of the ladies of an hospital connected with the Seminary ; and introduced him to his own patients in the city. He was thunder-struck when, on Sunday, he heard that Dr. Webster had been charged with such an offence, because he never knew or heard anything which could affect the character of that gentleman.

Dr. Jones was examined by Mr. Devlin to show the effects of chloroform. I have known ladies use language, when under the influence of chloroform, that they would blush to hear at any other time. They were most respectable ladies ; the language was awful ; where they got the language I don't know. [A laugh.]

Court—How do you account for this woman lying an hour and a half under the influence of chloroform.

Dr. Jones—To say the least of it, it was gross neglect. Medical men may leave a patient under the influence of chloroform, but then a nurse is placed beside them. A person may be as long under the influence of chloroform as this complainant, but it has not come to my knowledge.

Daniel Webster, a youth of about fourteen years of age, was examined by Mr. Devlin—I am a week in Montreal to-day; I came to Dr. Webster's to see my brother, Rowland. I don't know if the complainant was the woman I saw in Dr. Webster's surgery on the 22nd instant. I think it was she. About twelve o'clock she lay down on the sofa. About half past one my brother went down stairs with her. My brother and I were in the work-shop all the time she was on the sofa. The woman called him in once and he went in. Afterwards she went down stairs with him.

James Nichols, the husband of the complainant, here came into the box and produced the letter which he sent to the prisoner on hearing of the conduct of the latter towards his wife. It was as follows:—

September 22.

"Dr. Webster—I was much pained and surprised at the account of your vile conduct towards my wife while she was unconscious and helpless under your professional care. I have no words to express my sense of such conduct, and will await a legal exposure. In the meantime you need not proceed with the teeth, as my wife will not again place herself in your power. James Nichols.

The following was the reply:

"I am surprised to receive such a note from you. My reputation is too well established to be affected by anything. There is not the slighest foundation for such a complaint, as my laboratory was constantly open, with no less than three persons in it all the time, and as many more waiting in the room for their turn." The remainder of the letter was occupied with reference to $14 which the prisoner owed Mr. Nichols for gold leaf, and it went on to say that the writer expected better treatment from Mr. Nichols. The letter bore no signature.

Justice Alwin then charged the Jury, explaining carefully the case in regard to the offence the prisoner had to answer, namely, attempt to commit rape, or assault. The learned Judge explained that the charge of rape had been discarded in an early part of the trial; and impressed upon the Jury, with much solicitude, that the question they had to decide was whether, taking the evidence of the prosecutrix in all its particulars—taking into consideration the position which the prisoner held and the risk to be run if, under his circumstances, he should endeavor to violate the persons of females; whether, looking at everything, he was really guilty of an attempt to commit rape or even an assault. His Honor, in conclusion, remarked on the singular conduct of the husband of the female, and observed that it was extraordinary he sat down to write a letter on the night of Wednesday, waited an answer by mail on Thursday, and took no steps till Friday to this and other facts, the learned Judge concluded by advising the Jury, if they entertained a doubt to give the benefit of it to the prisoner at the bar.

The Jury retired at six o'clock, and after an absence of about three hours and a half, brought in a verdict of "Guilty of an attempt to commit rape, with a recommendation to mercy." The decision seemed to take the Court by surprise.

Mr. Devlin Are the jury individually agreed?

One of the jury, Yes.

Mr. Devlin Then to-morrow morning I will make a motion in arrest of judgment.—*Medical Journal.*

---

THE LATE TRIAL OF DR. WEBSTER FOR A CRIMINAL ASSAULT.—In our last issue we laid before our readers the details, as reported for the "Montreal Herald," of Dr. John Horatio Webster, who was charged with having committed a rape on the person of Mrs. Louisa Nichols, while the said Mrs. Nichols was under the influence of chloroform. We made no remarks on the case at the time, as we were desirous to ascertain what further effort of counsel on behalf of the prisoner would result in. Dr. Webster, however, being still confined in jail, and his sentence yet unpronounced, we propose entering into an examination of the medical questions connected with the evidence brought forward by the Crown, with the view of ascertaining whether or not the verdict, "Guilty of an attempt to commit rape, with a recommendation to mercy," brought in by the Jury, was clearly warranted by that portion of the evidence. Before taking up our pen we have endeavored to lay aside all prejudiced feeling against the prisoner; for we frankly confess that if we were to allow ourselves to be influenced by our pre-conceptions of his private character, we would not be at all inclined to treat him with favor. Our aim will be to analyse the medical portion of the evidence, and give an unbiassed opinion. We can not for a moment subscribe to the decision arrived at by many who are doubtful as to the guilt of the prisoner in this particular instance, viz., that he deserves punishment for other offences, and should therefore be allowed to suffer. Were justice to be administered in this manner—were men to be condemned for alleged crime, on insufficient evidence, merely because public report, attributed to them the commission of deeds similar or dissimilar to that for which they might at the time be arraigned before the legal tribunals of their country—it would certainly result in consequences the most disastrous. No individual under such a state of affairs would be safe from the malignancy of enemies. By means of a well organized combination, the character of an innocent person, as a preliminary step, could be very effectually injured, and then a certain definite crime being laid at his door, this very reputation could be employed to secure his conviction. The English practice of regarding a man as innocent until he be *proved* guilty, is, we believe, the greatest safeguard against injustice. Better that a guilty man should escape if proof were insufficient, than admit the principle of condemning an accused without adequate proof, because he is represented or known to have committed other offences deserving of punishment.

In entering on the consideration of the evidence brought at this trial, the first question that presents itself for solution is,—What was the anæsthetic administered? That this has an important bearing will be readily admitted when the effects produced by the inhalation of the two principal anæsthetics now in use—chloroform and ether—are compared with each other. It is too much the practice among medical practitioners to regard all anæsthetic agents as possessing identical properties. It is true that through the whole series of those carbon compounds which form the true as distinguished from the false anæsthetics, there is, in their effects on the human system, a certain amount of resemblance, but each one, nevertheless, has definite peculiarities which distinguish it from all the others. They differ widely, for instance, in anæsthetic power. Light Carburetted Hydrogen, Alcohol, the compounds of Methyle, and the Naphthas, are much feebler in this respect than the Ethers and compounds of Formyle. Aldehyde and Formic Ethers are substances that act powerfully in producing insensibility, but they give rise to so much irritation that it is impossible to employ them in practice. Carbonic Acid and Carbonic Oxide are powerful narcotic poisons, Bromide of Olefiant Gas, which is pleasant and agreeable while being inhaled, producing no irritation whatever, has been followed in a few hours by death when adminto animals. The primary effect of Ether and Chloroform when inhaled is the same—an irritant impression on the mucous membrane of the air passages, causing slight cough. This, however, soon subsides. In the case of Chloroform, when twenty to thirty minims have been administered, it is followed in a space of time, varying from ten seconds to two minutes, by symptoms of approaching unconsciousness. There is a feeling of buzzing or pulsation in the head, the brain becomes confused; the special senses are disordered; strange sounds are heard, and the color of objects altered. This is accompanied in some persons by very pleasurable sensations, and agreeable hallucinations; but in others by a feeling of suffocation, which is very distressing, and causes the patient to make strenuous efforts to free himself from the operator. Loss of consciousness succeeds, more or less complete, with quiet sleep, or sometimes unquiet sleep, with a tendency to incoherent talking and laughter. This state of insensibility remains for a period of five or six minutes, and when it passes off there exists either no recollection whatever or a very undefined notion of what has passed. When the dose has been one or more fluid drachms the effect is more powerful and rapid. Feelings of an agreeable character are soon followed by diminished sensibility, general numbness, mental obtuseness, drowsiness, complete loss of consciousness, and profound sleep. The eyelids droop; the pupils are dilated, though contractile, and roll upwards; the breathing is slow, often stertorous, and sometimes with frothing at the mouth; sensibility and the power of movement are quite lost and the muscles are in general universally relaxed, though in rare instances slight convulsive twitching of the face and limbs are observable. From the state of deep stupor or coma, the patient usually passes, for a short time, into a soft sleep, or dreamy drowsiness before fully awakening; but not unfrequently there is an immediate return to complete consciousness and the power of motion."

In the case of ether this primary effect is followed by symptoms of *general stimulation*. The pulse becomes rapid and the face flushed. The patient is exhilerated, sometimes quiet, but usually exhibiting considerable agitation of the muscular system, amounting in some, to convulsions. In a period, varying from two to ten or fifteen minutes, sleepiness is produced, and anæsthetization is known to have supervened by the closure of the eyes, the relaxation of the muscles, and the patient falling back apparently unconscious. "The mind, however, is not wholly inactive; for the individual often afterwards speaks of curious dreams or visions, which seem to him to have been of long duration, and which occasionally disagreeable or even fearful, are for the most part very much the reverse; and, altogether, the effects are so pleasing that a repetition of the process is frequently desired. But, with an increased influence of the vapor, a deep comatose sleep is induced, often attended with snoring, in which there is an entire loss of consciousness. If the agent is omitted as soon as the stupor appears, this state subsides as quickly as it was produced; and, though there may sometimes remain a momentary confusion of mind, and slight languor of body for a short time, occasionally perhaps a little headache or nausea, the subject of the process soon returns to his previous condition, as if nothing had happened. *In the period of excitement, it occasionally happens that the sexual function becomes the special seat of stimulation; and the delusions of the patient, or his dreams, may take a corresponding direction, and, even after the perfect return of consciousness, may remain impressed on the mind with the vividness of reality.*"* In addition to its power of abolishing sensibility, relaxing spasm and inducing a state of profound unconsciousness, ether has often a remarkable effect on the mucous tissues, *causing a great relaxation with increased discharge from their surfaces. This has been particularly noticed in the mucous membrane of the generative organs of woman*, and in that of the bronchial tubes. Now by comparing the effects of ether with those of chloroform, it will be perceived that they agree on two points only: 1stly. They produce a certain amount of irritation when first inhaled; 2ndly. They both, when a certain quantity is administered, induce a state of profound coma in which the patient becomes completely unconscious, and sensibility is entirely abolished. The manner, however, in which the patient is affected as he gradually becomes placed under the influence of each anæsthetic is markedly different; and there are phenomena attending the inhalation of the one which are not observable in that of the other. Chloroform appears to be a powerful and direct sedative to the nervous system, af-

* Wood's Therapeutics and Pharmacology, Vol. 1, p. 688.

fecting secondarily the circulation, and the respiration. Ether is primarily an excitant, and like other intoxicating agents is probably absorbed into the blood, by which means it comes into direct contact with the nervous system, the functions of which it first increases, then deranges, and finally depresses to a greater or less degree. But it is the peculiar effects which sometimes accompany the inhalation of ether that makes the question: What was the anæsthetic administered? of the highest importance in this trial. The fact that in many instances ether stimulates the sexual functions, producing erotic dreams, which the patient on becoming conscious, has great difficulty in believing to have been mere delusions and not actual realities; and that at times it produces also considerable discharges from the female generative organs, is of the highest medico-legal importance. In no work that we have had access to, are the same symptoms attributed to chloroform, and this agrees perfectly with our own experience; for in all the cases in which we have administered it ourselves, seen it administered by others, or assisted in its administration, never have we witnessed an indelicate action or gesture on the part of the patients, nor have we ever heard, during the period of inhalation or while the person was emerging from the state of anæsthetization, the utterance of obscene or improper language. Our observations of its effects, moreover, have been made on persons of different ranks of life. We have seen it administered in the large hospitals of Europe, to the lowest and most depraved persons, and in our own hospital we have operated on prostitutes while under its influence—we have seen educated gentleman and ladies, of refined manners, placed under its influence, and the results of our observations are such as we give above. The same opinion, we venture to say, is entertained by most, if not all the physicians in Canada who are accustomed to use it. We know it to be the opinion of Prof. Campbell, of this city, whose great experience of its effects, employing it, as he does, most extensively in the practice of midwifery, renders him in all probability, the greatest authority on such a question in the Province.

Two medical gentlemen were brought forward by the defense to give testimnoy to the effects of chloroform, and one of them (Dr. Jones) states: "I have known ladies use language, when under the influence of chloroform, that they would blush to hear at any other time. They were most respectable ladies; the language was awful; where they got the language, I don't know."

From what we have said, it will be seen we differ entirely from Dr. Jones, and we are confident, that if that gentleman will recall the circumstances under which he heard such language, he will find that the persons were etherized and not chloroformized. The second was Dr. W. Nelson, one of the oldest and most experienced physicians of the city. His testimony, which was intended to apply to chloroform, bears out our views. To prove that the inhalation of chloroform is capable of causing excitement of the sexual functions, and fixed delusions, he gives in illustration a case in which he administered ether. "I once operated on a woman who had a tumor. I got the loan of an apparatus *to administer ether.* I received it from Dr. Webster. *The patient took ether*, and I removed a tumor of seven pounds weight. The woman for two days held the opinion, though many of her neighbors were witnesses of the operation, that I abused her."

The second question that presents itself is:—Are there statements contained in the evidence which would indicate the particular anæsthetic employed?

During the trial it was apparently taken for granted that chloroform was administered. The defence admitted, whether or not by the direction of the prisoner we know not, that chloroform was the anæsthetic given by Dr. Webster; and endeavored to substantiate that all the prosecutrix affirmed was quite explicable by the effects of chloroform on the system.

There appears to have been an impression on the minds of all concerned that the effects of chloroform and ether are identical, and that all that was stated with regard to one applied with equal force to the other. This we consider to have been particularly unfortunate, for if it could be established that ether, or, what is a very favorite combination with American Dentists, chloroform and ether united, was the anæsthetic administered, it would go far to satisfy the minds of many medical men that Mrs. Nichols has been laboring under a delusion; a view they can not at present entertain, judging from what they believe to be the effects of chloroform.

Were it established beyond a doubt that ether was inhaled either simply or combined with chloroform it would serve, moreover, to account not only for the erotic feelings but also for the wet condition in which Mrs. Nichols found her underclothing. "When I went home," she says, "I perceived that my under-clothes were very wet. I became aware of this circumstance before I left the prisoner's office. I thought it was my own urine, and attributed it to the fact that I had been greatly pulled about." As we have stated above, ether has at times a relaxing effect on the mucous tissues, causing a profuse discharge from them, and that this effect is more manifest in the membrane lining the generative organs of females than that of any other portion of the body. This condition of her clothes has been considered by many persons as strong proofs of the guilt of the prisoner; but the explanation becomes easy on the supposition that ether was the anæsthetic. We are not aware of the same circumstance having been observed in connection with the administration of chloroform. So much then with regard to the anæsthetic. We will now proceed briefly to advert to a few points which seem to favor the view that Mrs. Nichols was laboring under a delusion, the result of the stimulating effect of the substance inhaled on the generative organs. In the first place she was in a condition which rendered her peculiarly susceptible to excitement of these organs on the suspension of the will, and, as a consequence, her dreams or delusions would take their complexion from the excited sexual function. She was, in other words, menstruating at the time of her visit to Dr. Webster's surgery.

Again, her recollection of the order in which events occurred, and the manner of their occurrence is somewhat indistinct. She states, for instance, in her evidence in chief, that when she first became partially conscious, she *saw* she was not in the chair, but on a sofa, and she also *saw* that the prisoner was sitting along with her on the sofa ; that she again became unconscious, and the next thing she recollects was that the prisoner was still sitting on the sofa beside her, and that he had his hand in an improper position, and had placed hers in the same. In cross-examination, however, she says : "I *felt* the prisoner's hand in an improper position upon my person, but *I did not see* it. *I did not see* my own hand in a similar position on his. *I felt but did not see* him sitting on the sofa beside me."

From these considerations then, coupled with the evidence of a non-medical character, which may be found in our last number, we are of opinion that the prisoner has been convicted on insufficient evidence. He may certainly be guilty, but *we* could not condemn him on such slight evidence.—*M. Jour.*

